# Efficiency data of intracellular recombinant protein delivery using cationic lipid coated silk fibroin particle

**DOI:** 10.1016/j.dib.2017.06.041

**Published:** 2017-07-01

**Authors:** Woo-Jin Kim, Bong-Soo Kim, Young-Dan Cho, Won-Joon Yoon, Jeong-Hwa Baek, Kyung-Mi Woo, Hyun-Mo Ryoo

**Affiliations:** Department of Molecular Genetics, School of Dentistry and Dental Research Institute, BK21 Program, Seoul National University, Seoul, Republic of Korea

## Abstract

This article presents data related to the research article “Fibroin particle-supported cationic lipid layers for highly efficient intracellular protein delivery” and focuses on the delivery efficiency aspects of the fibroin particle-cationic lipid complex (Fibroplex), including its fabrication and the intracellular delivery to the mouse skin tissue. We introduced a stable lipid-particle complex called “Fibroplex”, formed by loading cargo protein onto a silk fibroin spherical particle core complexed with cationic liposomes to address the intracellular recombinant protein delivery. This system exhibits cationic charge, which is advantageous for cellular uptake. The particle core is loaded with the cargo protein with high efficiency and shows long-term release in serum environment. Fibroplex can be formed simply by mixing the particle core and cationic liposome, and this spontaneous interaction does not cause any detrimental effects on the function of cargo proteins. Lipid-particle complex structure is stable over 10 days in the serum at 37 °C. Fibroplex was delivered at high efficiency to a wide variety of cells, including cancer cells and primary cell-lines. Also, Fibroplex loaded with two types of cargo successfully introduced them into the cytoplasm. Furthermore, Fibroplex shows successful intracellular delivery when injected with various cargo proteins such as GFP, HRP and Tyrosinase into mouse skin tissue as well as in vitro. The highlights of this article include: (1) Data for fabrication procedure of Fibroplex, (2) loading capacity, surface charge changes of Fibroplex, and (3) Intracellular delivery aspects of Fibroin in vitro and vivo.

**Specifications Table**

**Table:** 

Subject area	*Drug delivery, Biology, Material Sciences*
More specific subject area	*Biomaterial, Intracellular protein delivery, Lipid-particle complex*
Type of data	*Graph, Figure, Table*
How data was acquired	*Confocal laser Microscope, SEM, Zeta-sizer, Elisa, Western blot*
Data format	*Analyzed*
Experimental factors	*Cargo protein loading into fibroin particle*
Experimental features	*Intracellular delivery of lipid-particle complex in vitro and vivo*
Data source location	*Seoul, Republic of Korea*
Data accessibility	*Data is provided in the article*

**Value of the data**

• This work creates a deeper understanding of the direct recombinant protein delivery system using silk fibroin particle and lipid complex.
• The data in this article show how a design of experiment (DOE) approach can be used to produce efficient intracellular delivery of recombinant protein in various cell-lines and mouse skin model.
• The in vivo data in this article highlight the tissue uptake efficiency and distribution of the delivered cargoes - GFP, HRP and tyrosinase protein in the mouse dorsal skin layers.

## 1 Data

**Fig. 1:**
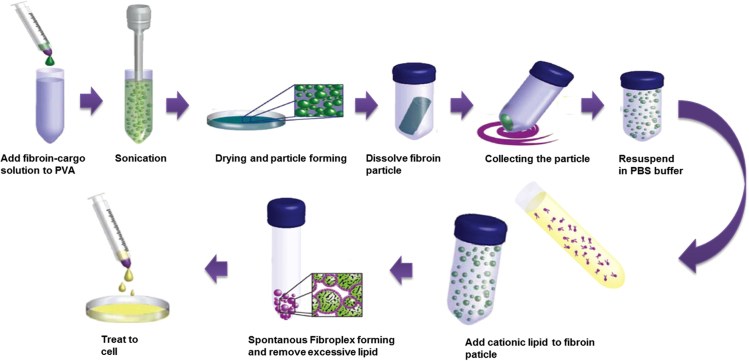
Schematic illustration of fabrication of Fibroplex. PVA(poly-vinyl alcohol) was solubilized in DDW and mixed with fibroin solution for 1:4 volume ratio. Sonication was performed by 30% amplitude suggested manufacturer(VC-130, Sonics, USA) for 30 s at 4 °C (for preventing heat induced damage). Cationic lipid (DOTAP:DOPE 1:1 wt%) were used for all experiments. Cationic lipids according to same weight ratio are mixed with fibroin particle and then vortexed for 30 s at room temperature. Fibroin particles and cationic lipids interact spontaneously, if incubated 30 min at room temperature. Excess lipid is removed via light centrifugation before treatment.

Silk fibroin is a natural fibrous protein with advantages for protein loading and delivery in vitro and vivo. However, presumably because of the super-negative charge of fibroin, the application of intracellular delivery was limited. Lipid-polymer complex is formed by cationic liposomes complexed to the fibroin particle core as referenced [1]. Briefly, fibroin particles are prepared per unit weight and then dispersed by weak sonication at 10% amplitude (VC-130, Sonics, USA) for 30 s in 4 °C PBS. After particle dispersion, cationic lipids according to each weight ratio are mixed with fibroin particle solution and then vortexed for 30 s at room temperature. Fibroin particles and cationic lipids interact spontaneously, if incubated for 30 min at room temperature. Excess lipid is removed via light centrifugation before use (Fig. 1).

**Fig. 2:**
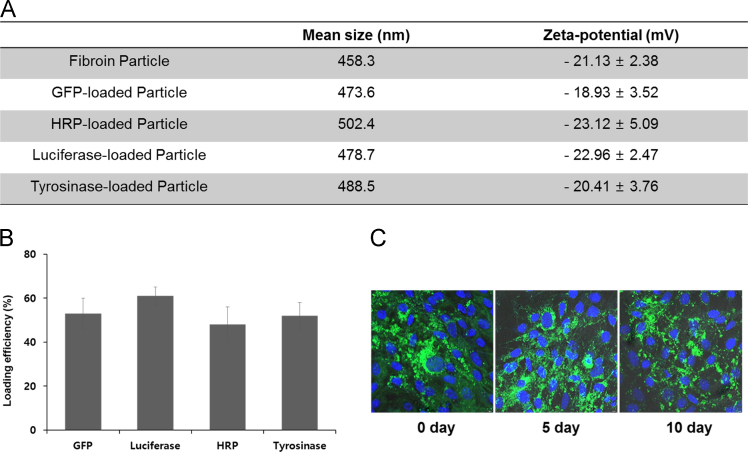
Surface charge, size, loading efficiency and stability of Fibroplex. A) fibroin solution were mixed with 150 nM protein solution. Mean size and surface charge measured by zetasizer. B) The loading efficiency was measured as the amount of protein contained in the supernatant when fabricating fibroin particles by ELISA. C) Structural stability of Fibroplex was measured by intracellular delivery after incubation at 37 °C for 10 days in serum. All error bars reflect the standard deviation of three independent biological experiments performed on different days. Scale =5 μm.

Fig. 2 shows the effect of various types of fibroin particle cargo proteins on particle size and surface electrophysical characteristics. Four proteins with different sizes and characteristics GFP, HRP, Tyr and Luc, respectively, showed similar size and zeta potential distributions (Fig. 1a). Fabrication of silk fibroin particle and loading cargo proteins were described previously [2]. Protein loading efficiency of fibroin particles was measured for four representative proteins - GFP, HRP, Luciferase and tyrosinase. Proteins showed an average introduction efficiency of 50–60% (Fig. 2b). Fibroplex displayed high structural stability for a long period of time at 37 °C. After Fib(GFP) containing 150 nM of GFP was formed, cells were treated with Fib(GFP) right away in one group (0 day), while other cells were treated with Fib(GFP) after it had been stored for five or 10 days at 37 °C; in all cases, Fib(GFP) was taken up by the cells(Fig. 2c).

**Fig. 3:**
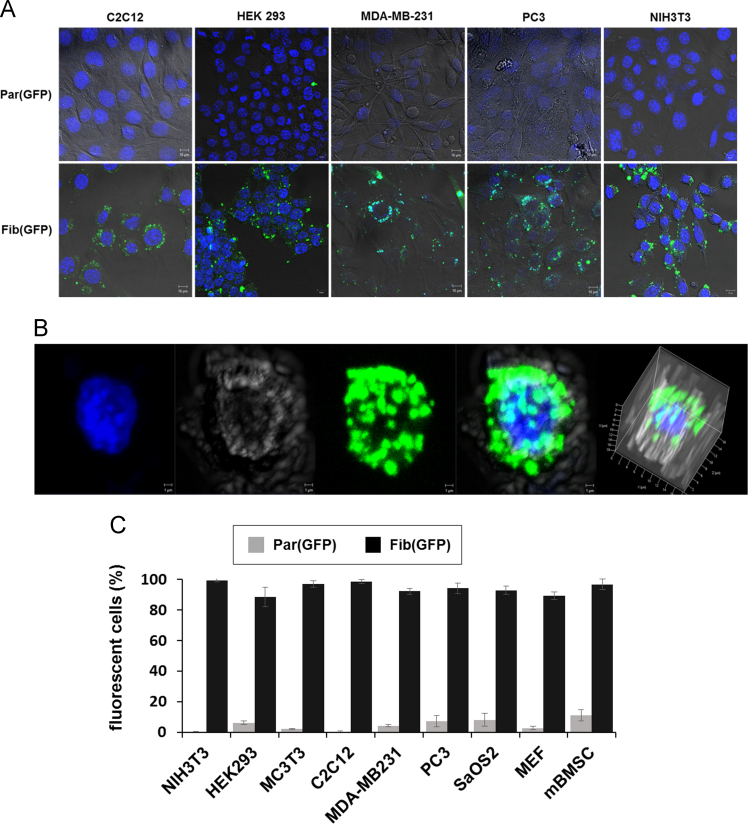
*in vitro* Intracellular efficiency of Fibroplex. A) 5 types of cell lines – C2C12, HEK293, MDA-MB231, PC3 and NIH3T3 were treated with Fib(GFP) and Par(GFP) for 48 h. B) Three-dimensional reconstruction of cells treated with fib(GFP) for 48 h. C) Flow cytometric analysis were performed after treated Fib(GFP) for 48 h. All error bars reflect the standard deviation of three independent biological experiments performed on different days. Scale=10 μm.

Fibroplex shows efficient intracellular delivery for various cell-lines. The same GFP protein was loaded (150 nM) into fibroin particles and fibroplex, but fluorescence was not observed in cell-lines treated with anionic fibroin particles [Part (GFP)] whereas high level of fluorescence was observed in all cell-lines treated with fibroplex [Fib (GFP)] (Fig. 3a). Introduced GFP was present mainly at high concentrations around the nucleus of the cell and uniformly throughout the cytoplasm (Fig. 3b). The FACS analysis conducted on nine different cell types, including cancer and primary cells, treated with Fib(GFP) and Par(GFP) showed that only approximately 1–7% of the cells that had been treated with Par(GFP) generated fluorescence, while the Fib(GFP)-treated cells showed much higher uptake rates. Specifically, 99% of NIH3T3 and C2C12 cells were positive, and MEF, mBMSC, MDA-MB231 and PC3 cells, which are primary or cancer cell lines, showed loading rates ranging from 88–98%, thereby confirming that particle-lipid complex structures can deliver EGFP into various cell types with high efficiency(Fig. 3c).

**Fig. 4:**
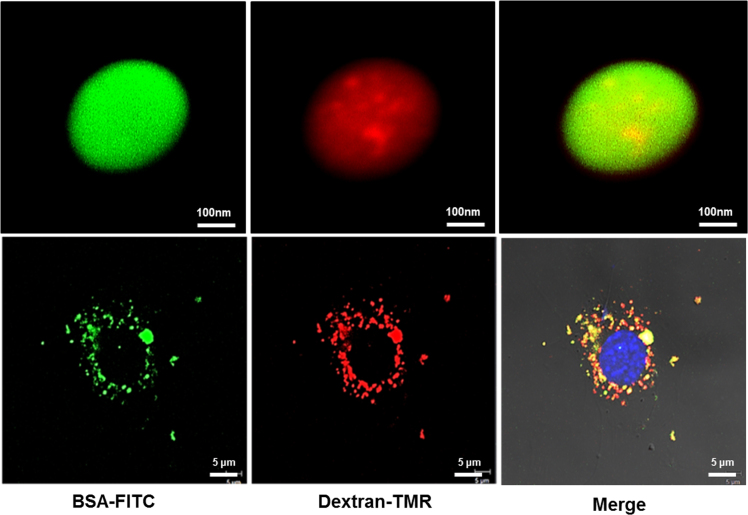
Simultaneous delivery of two cargoes by Fibroplex. Two fluorescence, green and red, are observed simultaneously in one fibroin particle (Upper panel). When cells are treated, red, green, and yellow fluorescence are observed in the same cell (Lower panel).

Also, Fibroplex loaded with two types of cargo successfully introduced them both into the cytoplasm. Fibroplex loaded with FITC-labeled BSA and TMR-labeled dextran penetrated the cell membrane, and different fluorescence was observed in the same cell (Fig. 4).

**Fig. 5:**
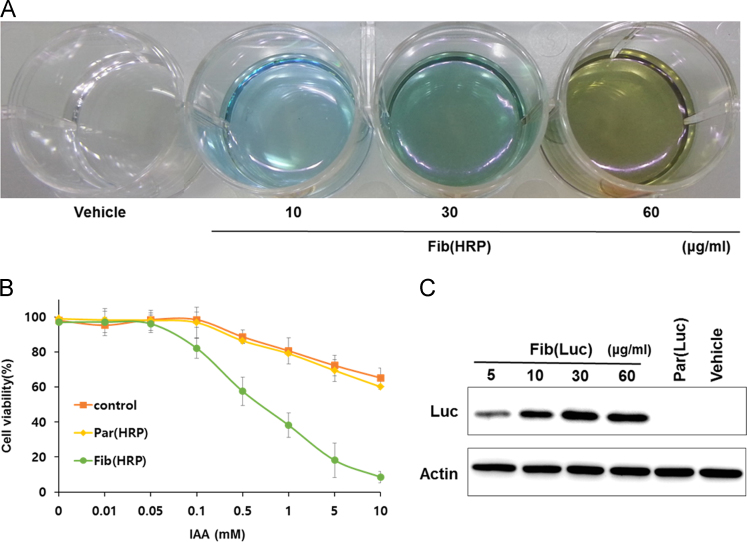
Concentration-related delivery and Intracellular Enzyme Activity of uptake HRP and luciferase. A) Fib(HRP) were treated with MC3T3-E1 cells incubated 24 well plate. 10 μg of Fib(HRP) contains 34 nM of HRP. After 24 h incubation, cells were washed three times for removal of residual particles and incubated with substrate for 30 min at 37 °C. B) B16-F10 cells were treated with Fib(HRP). Cytotoxicity was measured by MTT assay for each concentration. C) MC3T3-E1 cells were treated with Fib (Luc) at various concentrations, and the particles that were not internalized were removed after 48 h and the amount of luciferase was analyzed by western blot.

Fibroplex also effectively delivered HRP and luciferase into the cells. Cells treated with Fib(HRP) shows increased activity depending on treatment concentration (Fig. 5a). HRP forms active radicals that show cytotoxicity when treated with IAA(Indole-3-acetic acid) as a substrate[3]. When 150 nM of HRP was introduced into cells via Fibroplex, they showed higher cytotoxicity with increasing concentrations of treated IAA (Fig. 5b). After the treatment with fibroplex loaded with luciferase [Fib(Luc)], the amount of intracellular enzyme was increased according to the treatment concentration similar as in the case of Fib(HRP) treatment (Fig. 5c).

**Fig. 6:**
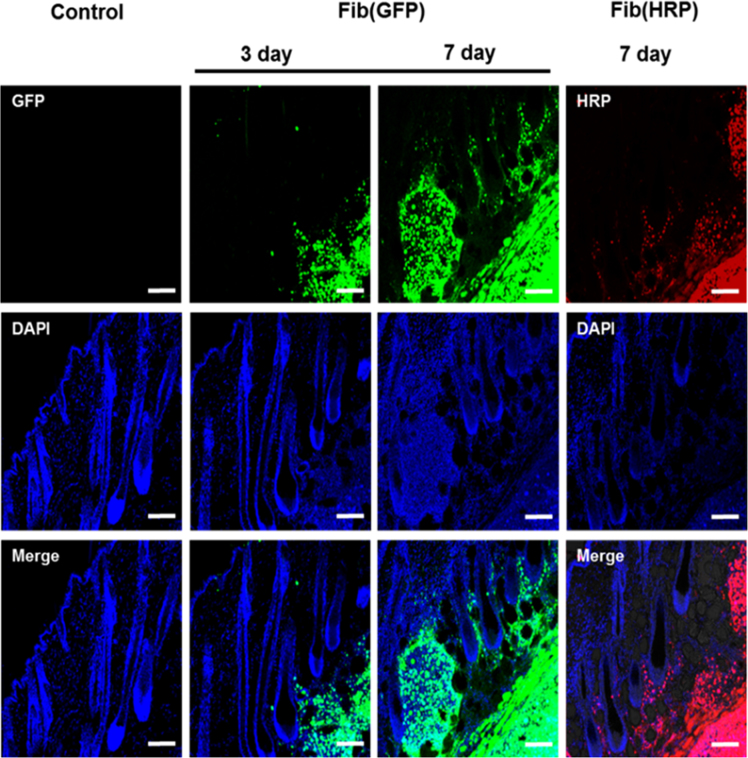
*in vivo* delivery of Fibroplex. Fibroplex loaded with GFP or HRP were subcutaneously injected into the dorsal skin of mice. At 3 and 7 days after injection, mouse were sacrificed and frozen sections of skin tissue was analyzed by confocal laser microscopy. Scale=50 μm.

To confirm whether the intracellular transport of Fibroplex occurs efficiently in vivo, we subcutaneously injected Fib(GFP) or Fib(HRP) into the dorsal skin of mice. We injected Fib(GFP) to observe the effects of transporting GFP into mouse skin cells. At 3 days after injection, we observed fluorescence in the lower layers of the dermis, and at 7 days after injection, fluorescence gradually increased in the dermis and epidermis. We did not observe such fluorescence distributions when we injected naïve GFP or Par(GFP) with the same vehicle, and no specific changes were observed in the major organs or mouse weight while the mice were being treated with Fibroplex (Fig. 6). HRP was also successfully delivered into skin tissue using Fibroplex. After injecting Fib(HRP), skin sections were assessed using dihydroethidium (DHE), a fluorogenic substrate for HRP. 7 days after injection, the skin tissues exhibited intense red fluorescence, and the distribution and enzymatic activity of HRP in skin tissue were similar to those of GFP (Fig. 6).

## 2 Experimental design, materials and methods

### 2.1 Materials

All chemicals were purchased from Sigma-Aldrich unless otherwise noted and were used as received. Polyvinyl alcohol (PVA, average MW 30,000–70,000, 87–90% hydrolyzed), rhodamine B, protease XIV, horseradish peroxidase (HRP), tyrosinase (Tyr) and all other chemicals used in the study were purchased from Sigma-Aldrich (Sigma-Aldrich, USA). Enhanced green fluorescent protein (EGFP) and TAT–EGFP fusion proteins were expressed according to methods described in previous reports. Fusion proteins were expressed in transformed Escherichia coli BL21 and purified using a nickel-resin affinity column (Sigma-Aldrich, USA). N-[1-(2,3-Dioleoyloxy)propyl]-N,N,N-trimethylammonium methyl-sulfate (DOTAP) and 1,2-dioleoyl-sn-glycero-3-phosphoethanolamine (DOPE), and N-(7-nitrobenz-2-oxa-1,3-diazol-4-yl)dioleoyl 1,2-Dioleoyl-sn-glycero-3-phosphoethanolamine (NBD-DOPE) were purchased from Avanti Polar Lipids (Avantilipid, USA). Ultrapure water from a Milli-Q system (Millipore, USA) was used throughout this research.

### 2.2 Cell line cultures

The MC3T3-E1 cells were maintained in ⍺-Minimum Essential Medium (⍺-MEM), and the HEK-293, NIH3T3 and C2C12 cells were maintained in Dulbecco׳s modified Eagle׳s medium (DMEM) with 10% heat-inactivated fetal bovine serum (10% FBS) supplemented with antibiotics. Mouse embryonic fibroblasts (MEFs) were isolated from E13.5 embryos, as previously described [36]. The MEFs were grown in DMEM/10% FBS, and cells from passages 3 to 5 were used.

### 2.3 Preparation of fibroin particles and Fibroplex

Fabrication of fibroin particle and loading the cargoes is based on previous reports [2], [4]. Stock solutions with a concentration of 500 µM in PBS buffer, pH 7.4, were first prepared and stored at −20 °C. Before loading, certain amounts of the stock solutions were added to the silk solutions to reach a drug:silk volume ratio of 1:9. After mixing, the solution was blended with the PVA solution following the steps described for the fibroin particle preparation. A 5 wt% polymer concentration and silk:PVA ratio of 1:4 was used to produce Fibroplex. Fibroplex were formed by cationic liposomes complexed to the fibroin particle core. In detail, fibroin particles are prepared per unit weight and then dispersed weakly sonicating at 10% amplitude (VC-130, Sonics, USA) for 30 s in 4 °C PBS. After particle dispersion, cationic lipids according to each weight ratio are mixed with fibroin particle solution and then vortexed for 30 s at room temperature. Fibroin particles and cationic lipids interact spontaneously, after incubating for 30 min at room temperature. Excess lipid is removed via light centrifugation before use. Cationic lipids (DOTAP) with 1:1 w/w PE(DOPE) were used in all further studies and were expected to be coated on the outer surface of the fibroin particle core. As a fluorescence label for cationic lipid membranes, 0.1 wt% 7-nitrobenzofurazan-labeled DOPE(NBD-DOPE) was used to study the membrane structure of Fibroplex. Scanning electron microscopy (SEM) images were obtained with a Hitachi S-4700 field-emission instrument. Dynamic light scattering (ELSZ 1000, Photal Otsuka electronics, Japan) was used to determine the hydrodynamic size and zeta potential of Fibroplex.

### 2.4 *in vitro* cellular delivery assay

The results of cellular internalization studies were assessed via confocal laser microscopy (CLM) and fluorescence-activated cell sorting (FACS). MC3T3-E1 cells were cultured in ⍺-MEM supplemented with 10% FBS and 1% penicillin/streptomycin. Cells (1.5×10^6^ cells/100 mm plate) were seeded the day before Fibroplex was added. Fibroplex and fibroin particles loaded with equal protein concentrations were added to the cell medium. After incubation at 37 °C for 12 h, the cells were washed three times with PBS containing heparin sulfate (40 µg/ml) to remove free particles. Then, the cells were incubated for an additional 24 or 48 h and either visualized with by CLM or trypsinized, centrifuged, re-suspended in PBS and analyzed via FACS. The actin staining was performed according to manufacturer׳s instructions. Briefly, after incubation with Fibroplex, cells were briefly washed, fixed with 2% formaldehyde, stained with rhodamine-phalloidin for 10 min, and then observed by confocal laser microscopy (CLM).

### 2.5 Cell growth inhibition assay

B16-F10 melanoma cells placed in 96-well plates (3000 cells/well) were incubated with HRP-loaded Fibroplex, fibroin particles or native HRP for 12 h; after being washed, the cells were exposed to different concentrations of IAA for 24 h. The half maximal inhibitory concentration was determined from the cell viability curve determined using MTT.

### 2.6 *in vivo* evaluation of Fibroplex uptake

All animal experiments were conducted after obtaining the approval of the Seoul National University Institute of Laboratory Animal Resources and Use Committee. Each of the C57BL/6 mice (Orient Bio., Kyungi, Korea), 6–8 weeks old, was subcutaneously injected with 150 µl of a Fibroplex saline solution, a fibroin particle saline solution, or a saline solution (control). After 3 or 7 days, the mice were sacrificed, and the skin and all major organs were excised for ex vivo imaging. Then, the frozen organs were embedded in a freezing medium, and cyrosections were prepared using a microtome. The tissue sections containing Fibroplex were stained with both DAPI (for nuclei) and dihydroethidium for HRP and then observed by CLM.
